# From Multi- to Single-Hollow Trimetallic Nanocrystals by Ultrafast Heating

**DOI:** 10.1021/acs.chemmater.3c01698

**Published:** 2023-11-06

**Authors:** Vanesa Manzaneda-González, Kellie Jenkinson, Ovidio Peña-Rodríguez, Olivia Borrell-Grueiro, Sergio Triviño-Sánchez, Luis Bañares, Elena Junquera, Ana Espinosa, Guillermo González-Rubio, Sara Bals, Andrés Guerrero-Martínez

**Affiliations:** † Departamento de Química Física, Universidad Complutense de Madrid, Avenida Complutense s/n, 28040 Madrid, Spain; ‡ EMAT, University of Antwerp, Groenenborgerlaan 171, B-2020 Antwerp, Belgium; § Instituto de Fusión Nuclear “Guillermo Velarde”, Universidad Politécnica de Madrid, José Gutiérrez Abascal 2, E-28006 Madrid, Spain; ∥ Departamento de Ingeniería Energética, ETSII Industriales, Universidad Politécnica de Madrid, José Gutiérrez Abascal 2, E-28006 Madrid, Spain; ⊥ Instituto Madrileño de Estudios Avanzados en Nanociencia (IMDEA-Nanoscience), Cantoblanco, 28049 Madrid, Spain; # Instituto de Ciencia de Materiales de Madrid, Consejo Superior de Investigaciones Científicas, Calle Sor Juana Inés de la Cruz 3, 28049 Madrid, Spain

## Abstract

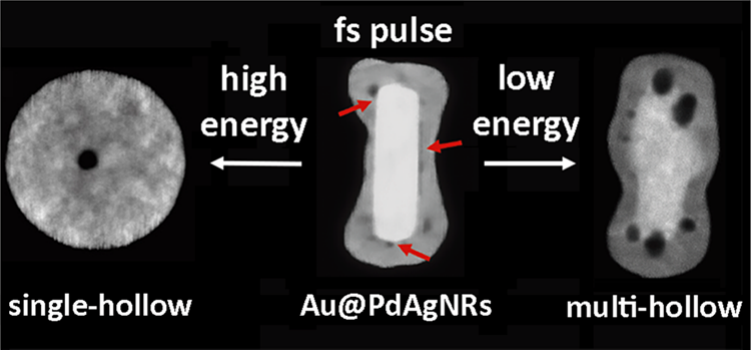

Metal nanocrystals (NCs) display unique physicochemical features that are highly dependent on nanoparticle dimensions, anisotropy, structure, and composition. The development of synthesis methodologies that allow us to tune such parameters finely emerges as crucial for the application of metal NCs in catalysis, optical materials, or biomedicine. Here, we describe a synthetic methodology to fabricate hollow multimetallic heterostructures using a combination of seed-mediated growth routes and femtosecond-pulsed laser irradiation. The envisaged methodology relies on the coreduction of Ag and Pd ions on gold nanorods (Au NRs) to form Au@PdAg core–shell nanostructures containing small cavities at the Au–PdAg interface. The excitation of Au@PdAg NRs with low fluence femtosecond pulses was employed to induce the coalescence and growth of large cavities, forming multihollow anisotropic Au@PdAg nanostructures. Moreover, single-hollow alloy AuPdAg could be achieved in high yield by increasing the irradiation energy. Advanced electron microscopy techniques, energy-dispersive X-ray spectroscopy (EDX) tomography, X-ray absorption near-edge structure (XANES) spectroscopy, and finite differences in the time domain (FDTD) simulations allowed us to characterize the morphology, structure, and elemental distribution of the irradiated NCs in detail. The ability of the reported synthesis route to fabricate multimetallic NCs with unprecedented hollow nanostructures offers attractive prospects for the fabrication of tailored high-entropy alloy nanoparticles.

## Introduction

In the era of nanotechnology, the art of manipulating metals at the atomic scale has proven to be essential for the development of advanced nanoscale materials. The fabrication of semiconductor chips, nowadays essential for computing, communication, or transportation systems, is probably one of the most paradigmatic examples.^1^ Noble metals have drawn significant interest due to their high chemical stability, even in the nanoscale, where most metals tend to rust and oxidize.^2,3^ Moreover, they are critical for the fabrication of nanostructures that are crucial to face challenges such as climate change commitments.^4−7^ Platinum, palladium, or iridium nanoparticles, and many of their alloys, are among the most active materials for the (electro)catalytic production of hydrogen, the element with the greatest potential to replace fossil fuels in energy storage, transportation, and use.^8−10^ In medicine, the strong optical characteristics of gold nanoparticles can be applied to develop sensors for early detection of various diseases, diagnoses, or cancer treatments.^3,11,12^

Nevertheless, obtaining valuable material from metals depends on our ability to transform them into nano-objects with defined shapes, sizes, and compositions. Colloidal synthesis routes, where metal nanocrystals (NCs) are usually dispersed in a liquid medium, are one of the most advantageous methods to produce metal nanoparticles with desired dimensions (from 1 nm to above 200 nm), morphological (spheres, cubes, etc.) and compositional characteristics.^13−15^ Moreover, they provide unprecedented control over the aggregation state of the metal NCs, a critical aspect of nanoparticle assembly in complex structured materials.^16−18^ In colloidal synthesis of noble metal nanoparticles, nucleation and growth of the NC occur through the reduction or decomposition at elevated temperatures (e.g., typically between 100 and 350 °C) of the metal precursors. Colloidal routes also present significant advantages for the synthesis of heterostructured multimetallic nanoparticles, in which the combination of different metals in the form of core–shell, Janus, or heterostructure can lead to novel or improved catalytic, electric, optical, and magnetic properties or enhanced corrosion resistance.^19−22^ Unfortunately, there are significant limitations for the synthesis of multimetallic NCs in the form of alloys, due to the distinct reduction potentials of different noble metals, which ultimately restrict their uniform coreduction into alloy nanoparticles.^23^ At the macroscale, metal alloy materials are frequently obtained by means of high temperatures that facilitate, for instance, the melting or sintering of different metal particles.^24^ Unfortunately, heat-assisted synthesis of alloy nanoparticles is less successful than in bulk because melting and sintering processes tend to favor their coalescence into larger particles and their reshaping into spheres (i.e., loss of dimensional, morphological, and aggregation state control), demanding the use of suitable substrates to prevent such issues.^25^

In this scenario, an ideal method of alloy nanoparticle synthesis would be to provide control over size, shape, and composition of colloidal synthesis routes with that delivered by high-temperature routes to obtain alloys. In practice, it has been demonstrated that colloidal nanoparticles of noble metals such as gold and silver can be heated well above their melting and boiling points while remaining in the dispersed state in solution.^26−33^ Specifically, gold and silver can strongly interact with light through the formation of localized surface plasmon resonances (LSPRs) at the interface between the NCs and the surrounding medium.^34,35^ Excitation of LSPRs with femtosecond laser pulses enables the deposition of a large amount of energy (peak power of 10^7^–10^12^ W/cm^2^) in the nanoparticle lattice within a few ps, at a rate that is typically higher than the cooling dynamics, which occurs at time frames ranging from few tens of ps to one ns.^36,37^ When bimetallic plasmonic heterostructures are excited with femtosecond irradiation, the temperature increase facilitates the mixing of metal atoms. Eventually, the formation of alloyed NCs can be observed, as has been successfully demonstrated for gold@silver and gold@palladium nanorods.^38,39^ Moreover, ultrafast heating of plasmonic nanoparticles mediated by ultrashort laser pulses can be implemented to improve their optical properties, enhance their catalytic behavior, or control their self-assembly behavior.^32,38,40^ In addition, the fast heating and cooling dynamics offer unique opportunities to obtain metastable structures that cannot be achieved by standard heating methods or colloidal synthesis routes, such as hollow gold NCs or partially alloyed anisotropic nanostructures.^37,39,41,42^ The introduction of a large number of voids and defects in alloyed nanocrystals is crucial for preparing high-entropy alloy nanoparticles because they can alter the electronic structure and density of states of the nanomaterials.^43^ These modifications can be advantageous in customizing the alloy’s properties for specific applications, such as enhancing its catalytic activity.^44^

In this work, we demonstrate the formation of anisotropic gold–palladium-silver NCs (MH AuPdAg NCs) with multiple holes in the structure via a synthesis strategy that combines seed-mediated growth of colloidal gold@palladium–silver nanorods (Au@PdAg NRs) with low fluence femtosecond laser pulses. The success of obtaining such NCs relies on the use of Au NRs as the core and the presence of Ag ions during the growth of the Pd shell, which leads to the formation of multiple small voids at the Au–PdAg interface during the shell growth. Subsequently, femtosecond laser irradiation produces the coalescence of these holes into multiple larger cavities. Larger laser fluences lead to the formation of fully alloyed single-hollow AuPdAg nanospheres (SH AuPdAg NSs), where the final dimension of the cavity is proportional to the Ag concentration. These observations were validated by high-angle annular dark-field scanning transmission electron microscopy (HAADF-STEM), energy-dispersive X-ray (EDX) tomography, and X-ray absorption near-edge structure (XANES) spectroscopy. Thereby, the presented research highlights the potential of combining colloidal synthesis methods of multimetallic NCs with laser irradiation to fabricate unique multimetallic nanostructures.

## Results and Discussion

**Figure 1:**
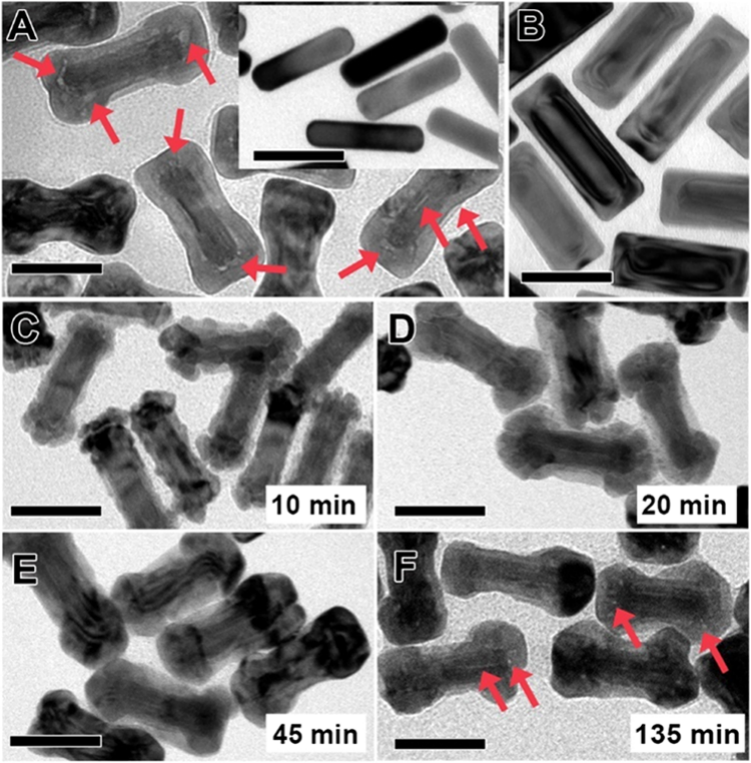
Synthesis of hollow Au_13_@Pd_76_Ag_11_ NRs. (A) Low-magnification TEM image of Au_13_@Pd_76_Ag_11_ NRs (inset shows the Au NRs used as seeds during the coating process). (B) TEM image of Au@Pd NRs (absence of Ag). (C–F) TEM images at different times during the growth process of Au_13_@Pd_76_Ag_11_ NRs: 10 min (C), 20 min (D), 45 min (E), and 135 min (F). Red arrows indicate the presence of voids. Scale bars: 50 nm.

The synthesis of colloidal anisotropic MH AuPdAg NCs described herein demands simultaneous control over several critical aspects: composition, anisotropy, and concentration and size of voids. Among existing synthesis technologies, seed-mediated growth routes provide optimal tunability of the nanoparticle shape, anisotropy degree, size, and composition.^14,16,21,45,46^ In practice, it relies on a multistep approach where the nucleation and growth processes can be precisely controlled by separating them in space and time. This approach effectively suppresses subsequent nucleation phenomena that often result in limited control over the size and shape. One of the most relevant cases is represented by the growth of gold nanorods (Au NRs), where size, shape yield, and anisotropic degree can be optimized when the symmetry breaking and anisotropic growth stages are separated in time and space (i.e., optimal experimental conditions for nucleation, symmetry breaking, and anisotropic growth are different in most cases).^47^ For these reasons, we aimed to use Au NRs as templates to obtain anisotropic MH AuPdAg NCs (Figure 1). For instance, the seed-mediated growth method has been combined with galvanic replacement methodologies to achieve both open and closed hollow multimetallic NCs.^48−53^

As an alternative methodology, we have developed a two-step synthesis approach where the first step relies on the core–shell Au@PdAg NC synthesis, followed by a secondary irradiation step with fs-laser pulses. In the first step, the growth of a PdAg shell onto Au NRs (18 ± 1 nm in width and 66 ± 4 nm in length) results in voids at the interface between the core and the shell (Figure 1A). The synthesis procedure involves the use of cetyltrimethylammonium bromide (CTAB) as the colloidal stabilizer and shape-directing agent and ascorbic acid to reduce Pd^2+^ and Ag^+^ on the Au NR surface (which acts as a seed for the heterogeneous nucleation of the PdAg shell). The resulting core–shell Au@PdAg anisotropic nanocrystals were obtained with increasing dimensions of 30 ± 3 nm in width and 82 ± 7 nm in length, with a slightly thicker diameter on the tips (39 ± 3 nm) (Figure 1A). The presence of both Pd and Ag was confirmed by EDX spectroscopy, which revealed final experimental concentrations of Ag, Pd, and Au of 11, 76, and 13%, respectively (Au_13_@Pd_76_Ag_11_ NR). The growth of the PdAg shell was also reflected in the plasmonic properties of the systems (Figure S1). The narrow plasmon band of Au NRs became significantly broad and blue-shifted after the deposition of Pd and Ag, due to the weaker plasmonic features of Pd, as confirmed by the calculated optical spectra of Au NRs and Au@Pd NCs performed by FDTD (finite differences in the time domain) simulations (Figure S2).^54^

Analysis of the TEM images suggests that voids, which can be observed as lower contrast areas around the Au NR seeds (as indicated by red arrows in Figure 1), are formed mainly at the interface between the Au NR and the PdAg shell. Notably, the formation of voids seems to be completely suppressed in the absence of Ag, and a homogeneous Pd shell with cuboidal morphology is obtained (31 ± 3 nm in width and 80 ± 8 nm in length, Figure 1B), in agreement with previous results reported in the literature for Au@Pd NC grown in the presence of CTAB.^55^ Interestingly, we observed the formation of corrugated nanocrystals, without any holes, in the presence of silver when CTAC (cetyltrimethylammonium chloride) was used as the stabilizing surfactant (Figure S3). The qualitative explanation can be attributed to the enhanced stabilization of Pd atoms in the presence of chloride because of lower polarizability compared to bromide counterions.^56^ Therefore, chloride counterions make Pd atomic diffusion more difficult, preventing the formation of vacancies or interstitial atoms.

To better understand the formation of voids, the evolution of the PdAg shell at different times during growth (Figure 1C–F) was further investigated by TEM. After 10 min of growth, the formation of an irregular shell was observed, characterized by the presence of grooves (25 ± 3 nm in width and 79 ± 8 nm in length, Figure 1C). Structures with similar dimensions were noticed at 20 min into the growth process (25 ± 3 nm in width and 79 ± 8 nm in length, Figure 1D), although a marked increase of the diameter at the tips became evident, i.e., from 30 ± 3 to 37 ± 3 nm. At this stage, the nanocrystal dimensions are close to those of the final Au_13_@Pd_76_Ag_11_ NRs. Indeed, at reaction times between 45 and 135 min, the size of the nanoparticles remains practically invariable (30 ± 3 nm in width and 80 ± 7 nm in length, 38 ± 3 nm in diameter at the tips; Figure 1E,F). However, important changes occured in the shell morphology; in particular, the grooves apparently disappeared, leading to a smoother surface. We hypothesize that the voids emerge during this stage, either as vacancies in the metal lattice or as some interstitial atoms from the medium.^57^ Finally, small voids start to appear after 135 min of growth (Figure 1F), and after 180 min, they are clearly visible (Figure 1A).

Based on the observed evolution of the PdAg shell during the growth process, where a groovy intermediate structure is formed and later filled, the presence of voids in the final structures might be explained by the appearance of some vacancies or interstitial atoms during this stage. Then, those defects and/or inclusions can migrate, getting trapped in the Au–PdAg interface and producing larger voids.^58,59^ Indeed, the formation of intermediate groove surfaces was not observed during the synthesis of Au@Pd NRs (Figure S4) in the absence of Ag, and those structures exhibited no voids. Hence, it is evident that the presence of silver is critical for both effects, the appearance of the wrinkles and the void formation, strongly suggesting that they are related. Moreover, it is well-known that Ag ions are capable of stabilizing Pd high index facets (via an underpotential deposition mechanism) and, thereby, induce the growth of intricate structures such as those formed during the initial stages of the synthesis process.^60,61^

To enhance our understanding of the electronic and atomic structure of the nanocrystals, XANES measurements were conducted on both the Au_13_@Pd_76_Ag_11_ NRs and Au@Pd NRs samples at the Au L_3_-edge (Figure S5).^62^ These measurements revealed identical spectra, indicating an unchanged white line intensity. This suggests that there is no distortion in the electronic states within the atomic structure related to the presence of voids. In contrast, both spectra exhibited a less intense white line compared to that of the Au foil. This reduction may be attributed to the presence of Pd, which enhances the filling of the Au d-band through the electron transfer from Pd to Au.

**Figure 2:**
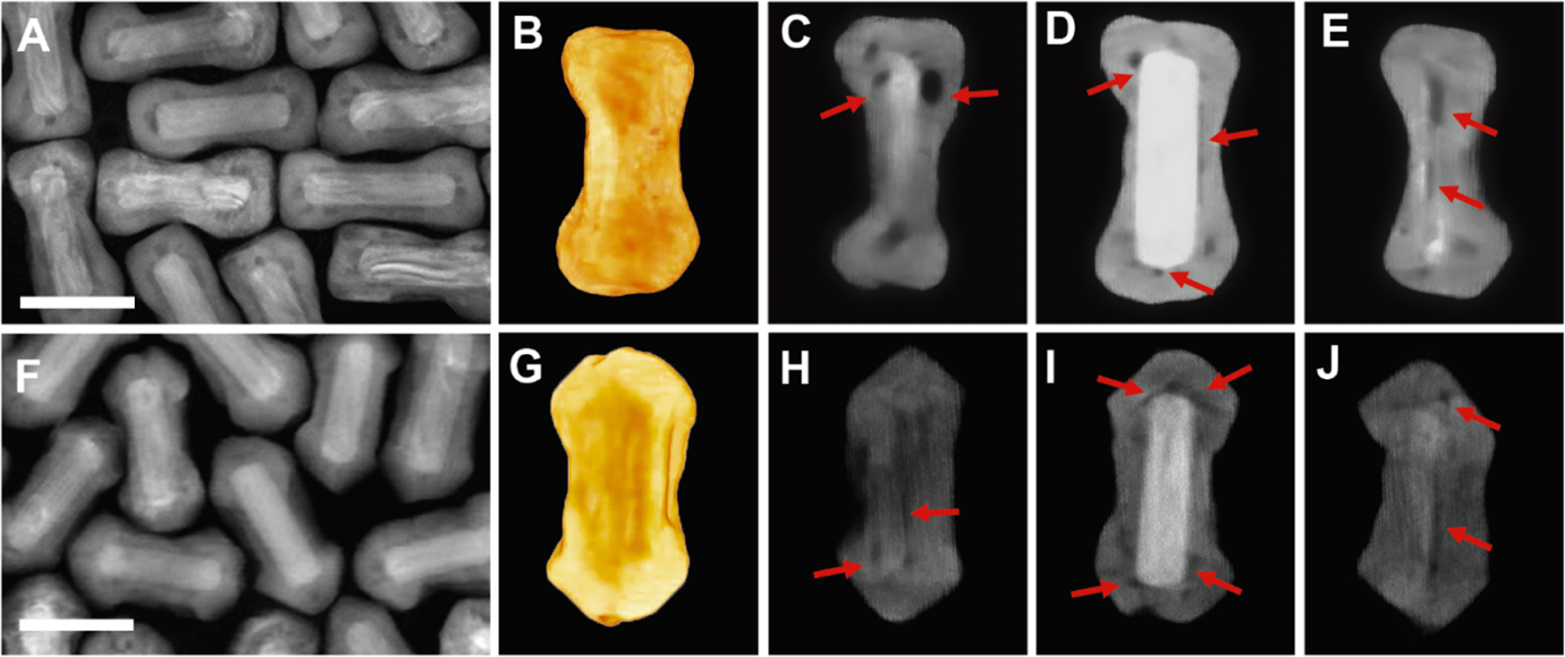
Effect of Ag on the formation of Au@PdAg NRs with voids. Characterization of Au_13_@Pd_76_Ag_11_ NRs (A–E) and Au_12_@Pd_69_Ag_19_ NRs (F–J) via HAADF-STEM (A, F) and HAADF-STEM tomography (B, G: 3D visualization of the HAADF-STEM tomography; C–E and H–J: the longitudinal sections through the middle of the 3D reconstruction) provided detailed information about the NC morphology and location of the voids. The red arrows indicate the presence of voids. Scale bars: 50 nm. Nanoparticle dimensions: 28 × 79 nm^2^ (B–E) and 30 × 78 nm^2^ (G–J).

From a single projection of the core@shell structures, the voids appear the most abundant at the Au–Pd interface. However, due to Au’s higher atomic number compared to Ag and Pd, TEM and STEM imaging of the structures is dominated by the Au core. Therefore, the Pd shell structure and the abundance and distribution of interfacial voids are only visible along the projected interface, which is also susceptible to the orientation of the deposited nanostructure. Thus, we characterized the complex multimetallic nanostructure in three-dimensional (3D) using electron tomography in high-angle annular dark-field scanning transmission electron microscopy (HAADF-STEM) mode to gain a clear understanding of void abundance and distribution throughout the entire Au_13_@Pd_76_Ag_11_ NRs structure (Figure 2). HAADF-STEM micrographs clearly revealed many voids in the PdAg shell (Figure 2A), mostly concentrated at the Au–PdAg interface. The HAADF-STEM tomography reconstruction allowed us to confirm the formation of a smooth PdAg shell with a cuboidal morphology (Figure 2B and Movies S1 and S2). Moreover, it was possible to locate the position of large voids in the longitudinal sections through the middle of the 3D reconstruction (Figure 2C–E and Movie S3), which revealed 5–10 nm voids with pseudospherical morphologies.

For Ag contents of 5%, we did not observe significant void formation (Figure S6); however, as the content increased from 11 to 19% (12% of Au and 69% of Pd as revealed by EDS analysis, Au_12_@Pd_69_Ag_19_ NRs) significantly impacts the Au@PdAg NRs nature, including the void occurrence (Figure 2F–J and Movies S4–S6). For instance, it partially hinders the formation of the PdAg shell, especially on the Au NR side, which was found to adopt a concave morphology. In addition, at the tip of Au_12_@Pd_69_Ag_19_ NRs, the PdAg shell seems to adopt a tetrahedral geometry (Figure 2G and Movie S4). More significant is, however, the fact that the dimensions of the voids seemed to be reduced to about 3 nm (Figure 2F–J). In the case of Ag contents exceeding 25%, we observed turbidity in the colloidal reaction solutions, indicating incomplete solubility of the metal precursors and impeding the use of such a compositional range.

The next step in the production of anisotropic MH AuPdAg NCs relied on the irradiation of the presynthesized Au_13_@Pd_76_Ag_11_ and Au_12_@Pd_69_Ag_19_ NRs with femtosecond (fs) laser pulses to induce the coalescence of the voids into larger cavities. Ultrashort laser pulses were chosen due to their ability to induce ultrafast heating of metal NC lattices. This can be understood if we consider that fs pulses deposit their energy on the nanoparticle lattice in a very short time (a few ps), much shorter than that required for the energy transfer to the surroundings (several hundred of ps).^26,27,63−67^ Hence, a nearly adiabatic heating process occurs, and the NC temperature can easily reach the melting point. Such an ultrashort and localized heating generally leads to an increase in the diffusion of the metal atoms, inducing controlled NC reshaping, typically toward thermodynamically stable morphologies (i.e., spheres).^34,38,39,44,68−70^ However, the out-of-equilibrium nature of pulsed laser-induced heating can also facilitate the production of metastable nanostructures that cannot be obtained via standard synthesis methods, as we have recently demonstrated for the case of partially alloyed Au@Ag NRs with rice-like morphology.^41^ Therefore, we hypothesized that femtosecond-pulsed laser irradiation of the synthesized Au@PdAg NRs containing small voids could promote their migration and coalescence into well-defined hollow nanostructures in a thermally activated process that reduces the internal surface area and, therefore, is thermodynamically favored. Of course, some reshaping of the elongated nanoparticle is also to be expected.

**Figure 3:**
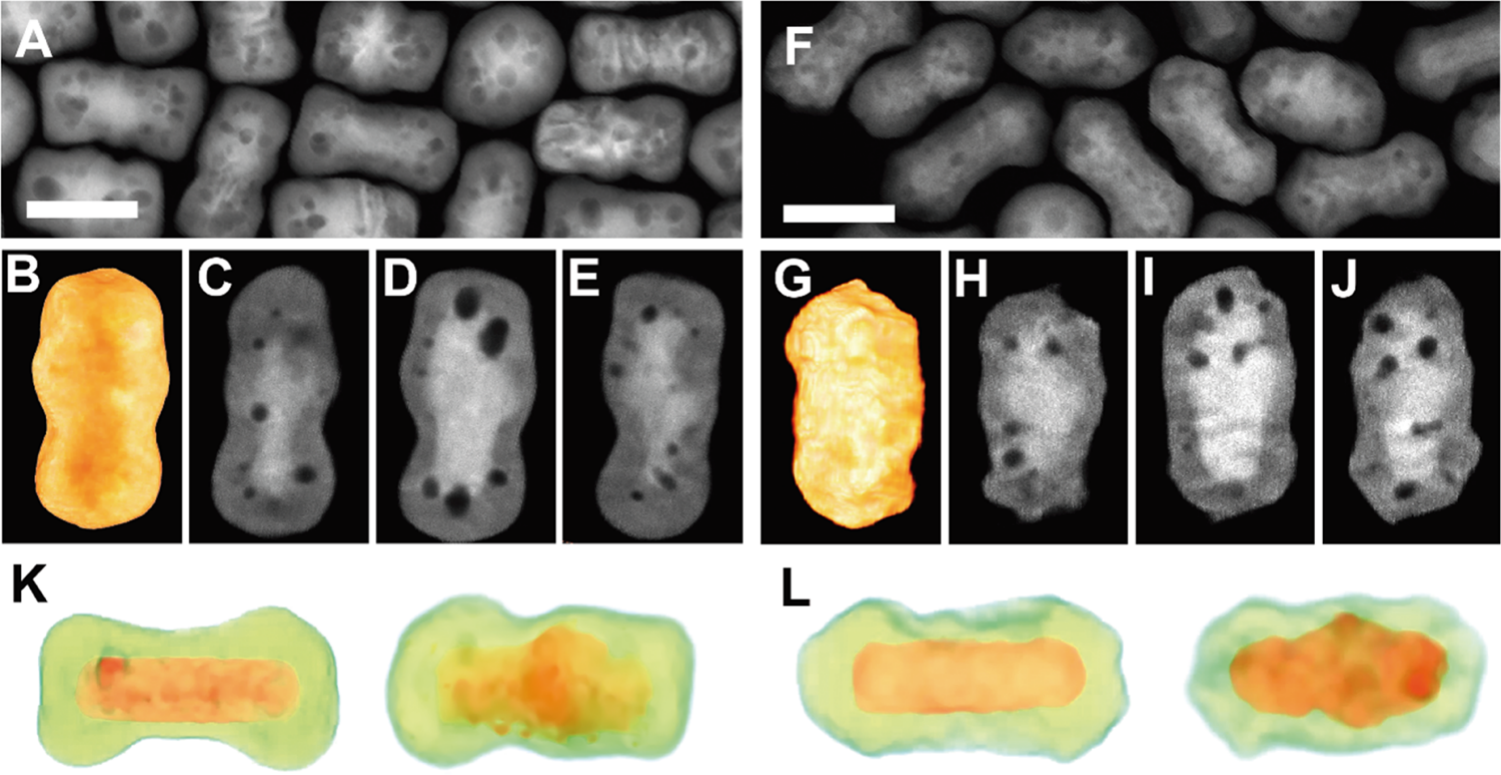
Irradiation of Au@PdAg NRs with 800 nm 50 fs-pulsed 1 kHz rep rate Ti:sapphire laser. HAADF-STEM images (A, F) and HAADF-STEM tomography reconstructions (B, G: 3D visualization of the HAADF-STEM reconstructions; C–E and H–J: the longitudinal sections through the middle of the 3D reconstruction) of Au_13_Pd_76_Ag_11_ (A–E) and Au_12_Pd_69_Ag_19_ (F–J) NRs irradiated for 25 min irradiation with fs-laser pulses at a fluence of 10 J/m^2^. (K, L) 3D visualization of the EDX tomography reconstructions of a nonirradiated (left) and an irradiated (right) Au_13_@Pd_69_Ag_11_ NR and Au_12_@Pd_69_Ag_19_ NR. (Au:red, PdAg:green). Scale bars: 50 nm. Nanoparticle dimensions: 39 × 77 nm^2^ (B–E, K right), 28 × 79 nm^2^ (K left), 36 × 70 nm (G–J, L right), and 30 × 78 nm^2^ (L left).

The proposed hypothesis was investigated by irradiating the as-grown Au_13_@Pd_76_Ag_11_ NRs with a 800 nm 50 fs-pulsed 1 kHz re. rate Ti:sapphire laser (Figure S1). The pulse fluence was found to be the critical parameter to induce the growth of small voids into larger cavities while minimizing the reshaping of the NRs into spheres. In this sense, irradiation with a pulse fluence of 10 J/m^2^ for 25 min was sufficient to maintain the anisotropic morphology for most of the structures (ca. 70%), albeit with a slight reduction of the average length, i.e., from 82 ± 7 nm to 69 ± 6 nm, and a concomitant increase of the width at the central region of the NRs, from 30 ± 3 to 40 ± 5 nm. More importantly, the irradiation produced some large cavities with dimensions of up to 15 nm and a quasi-spherical shape (Figure 3A–E and Movie S4). In all likelihood, those large cavities are formed due to the coalescence of the small voids, which almost disappear after the irradiation.

Similar results were obtained in the case of Au_12_@Pd_69_Ag_19_ NRs, where the cavity dimensions increased, but most of the irradiated NRs (ca. 70%) maintained the anisotropic morphology. However, the boost in cavity size was lower than that observed in the case of the irradiated Au_13_@Pd_76_Ag_11_ NRs (i.e., sizes were mostly below 10 nm, Figure 3F–J and Movie S5). This phenomenon can probably be related to the presence of a lower concentration of voids in the as-synthesized Au_12_@Pd_69_Ag_19_ NRs (Figure 2). Besides the observed growth in cavity size after the laser irradiation experiments, the length of Au_12_@Pd_69_Ag_19_ NRs decreased from 84 ± 4 to 76 ± 7 nm, and the width increased from 31 ± 3 to 34 ± 5 nm. It is worth noting that the irradiation of Au@Pd NRs with 10 mJ/m^2^ fs laser pulses does not induce the appearance of cavities in the NCs, as expected. This result supports the hypothesis that the cavities observed in the irradiated Au@PdAg NRs arise from the coalescence of the small voids present in the as-synthesized Au_13_@Pd_76_Ag_11_ and Au_12_@Pd_69_Ag_19_ NRs.

**Figure 4:**
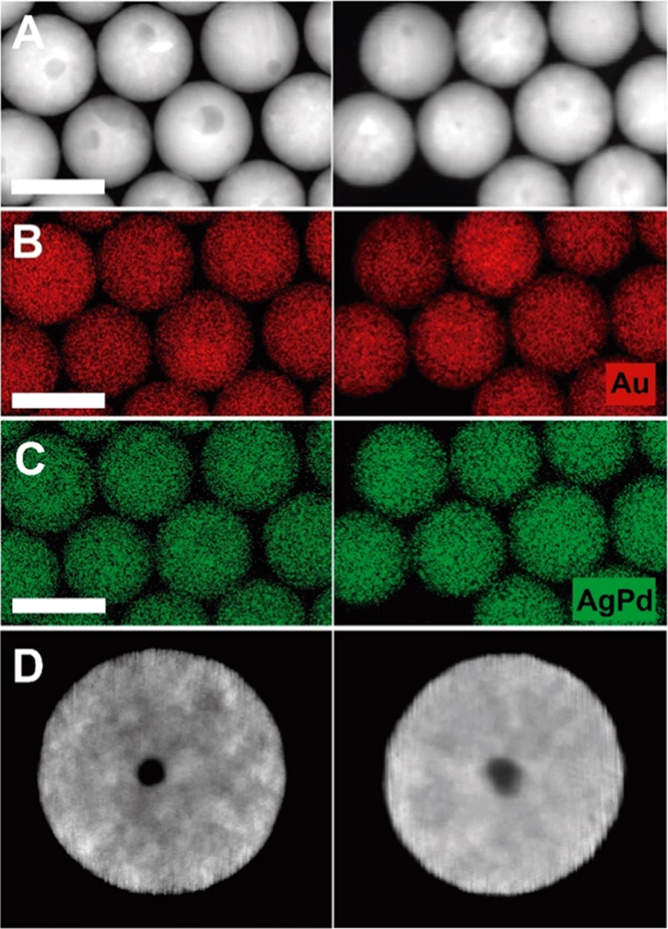
Synthesis of SH AuPdAg NSs via excitation of Au@PdAg NRs with 800 nm 50 fs-pulsed 1 kHz rep rate Ti:sapphire laser. HAADF-STEM images (A), quantified EDX maps (B, C), and longitudinal sections through the middle of the 3D reconstruction (D) of Au_13_Pd_76_Ag_11_ (left) and Au_12_Pd_69_Ag_19_ (right) NRs irradiated for 25 min irradiation with fs-laser pulses at a fluence of 70 J/m^2^. The initial Au@PdAg NRs were transformed into alloyed spheres (where Au, Pd, and Ag atoms are homogeneously distributed) containing a single cavity. Scale bars: 50 nm. Nanoparticle dimensions: 51 (D left) and 53 nm (D right).

To further understand the effect of the femtosecond-pulsed laser irradiation on the elemental distribution, we employed EDX tomography (Figure 3K,L). At the utilized pulse fluence, the heterostructure nature is maintained, although accompanied by a partial mixing of the constituents, as revealed by EDX tomography reconstructions (Au, Pd, and Ag are highly miscible; Figure S7 and Movies S13–S16).^71^ This effect suggests that the energy required to activate the cavity growth is lower than that for alloying Au with PdAg, which is compatible with our hypothesis of cavities formed as clusters of vacancies or interstitial atoms. However, one of the most appealing features of fs-pulsed laser irradiation is the possibility to finely tune the morphology and element distribution of multimetallic NCs through the applied pulse energy.^41^ Here, an increase of the pulse fluence to 70 J/m^2^ of the synthesized Au_13_@Pd_76_Ag_11_ and Au_12_@Pd_69_Ag_19_ NRs was sufficient to induce a total reshaping into spheres of 52 ± 4 nm and 50 ± 3, respectively, as well as to facilitate complete alloying (Figure 4A–D and Movies S17–S24). When high pulse fluences are used, a larger amount of energy absorbed by the NC electronic system is transferred to the lattice, which enhances atomic diffusion. Thereby, it is possible to promote the morphological transitions into more thermodynamically stable shapes (i.e., sphere) and redistribution of the different metal constituents observed for the investigated Au@PdAg system.^34,40,41,70^

It is remarkable that the cavities remain even under these aggressive irradiation conditions, which indicates that they are very stable. However, their number was dramatically reduced after irradiation so that each particle contained a single hole typically located at a slightly eccentric position. This effect can be observed in the transversal sections taken through the 3D tomography reconstructions, where the cavity site is slightly displaced from the NC center (Figure 4D and Movies S19 and S23). It was also noticeable that the cavity size was also somewhat reduced after fs-pulsed laser irradiation, resulting in cavities with an average size of ca. 10 and 5 nm for Au_13_@Pd_76_Ag_11_ NRs and Au_12_@Pd_69_Ag_19_ NRs, respectively. This latter result is compatible with the migration of some vacancies/atoms to the outer surface of the nanoparticle, where they are annihilated/released. Notably, no cavity formation was observed in the case of Au@Pd NRs, pointing out the importance of Ag for the formation of MH AuPdAg NCs and SH AuPdAg NCs (Movies S25–S35).

## Conclusions

In summary, we have envisaged a synthetic methodology to unlock the fabrication of multimetallic NCs with intricate hollow structures and anisotropic morphologies. The key aspect that opens access to nanoparticles containing several metals and cavities is the combination of colloidal growth routes and femtosecond-pulsed laser irradiation. The former facilitates the controlled formation of heterostructured anisotropic nanocrystals, while the latter enables the emergence of well-defined hollow nanostructures with different degrees of alloying. The proposed route was demonstrated for a Au–Pd–Ag system, where Au NRs were synthesized and overgrown in the presence of Pd and Ag to obtain a core–shell Au@PdAg nanostructure. Notably, the coreduction of Pd and Ag led to a shell containing small cavities, as revealed by TEM and confirmed by HAADF-STEM tomography reconstructions. Subsequently, the irradiation of Au@PdAg NRs with 800 nm 50 fs Ti:sapphire laser pulses at different fluences allowed us to induce shape and hollow degree modifications. For instance, MH AuPdAg NCs were obtained at 10 J/m^2^, where the Au NR core appeared to be partially alloyed with the PdAg shell, according to EDX tomography reconstructions. The deliberate introduction of numerous voids and defects into partially alloyed nanocrystals by ultrafast pulsed laser irradiation potentially plays a significant role in the production of high-entropy alloy nanoparticles. These features have the potential not only to increase the surface area of the nanoparticles but also to modify the reactivity of metals and their electronic structure, thereby potentially enhancing the catalytic efficiency and selectivity. At high fluence, 70 J/m^2^, the energy deposited on the NC lattice induced complete Au@PdAg NR reshaping into SH AuPdAg NCs. In this case, a homogeneous distribution of Au, Pd, and Ag was noticed, indicating the formation of hollow alloy NCs. Thereby, we have successfully shown a wet-chemistry approach to synthesize multimetallic NCs with unprecedented hollow structures based on seed-mediated routes and ultrafast heating with fs-laser pulses. This strategy should be applicable to synthesize high-entropy alloy nanoparticles, including other metals, such as platinum or even copper, provided that core–shell or core–multishell metal NCs are irradiated with laser pulses. Collectively, the presence of voids and defects in multimetallic alloy nanoparticles has the potential to make them highly effective catalysts.

## Experimental Section

### Chemicals

All starting materials were used without further purification. Cetyltrimethylammonium bromide (CTAB 99+%,), cetyltrimethylammonium chloride (CTAC 99.0%), sodium borohydride (NaBH_4_, 99%), Hydrogen tetrachloroaurate trihydrate (HAuCl_4_·3H_2_O, ≥ 99.9%), sodium tetrachloropalladate (Na_2_PdCl_4_, 98%), silver nitrate (AgNO_3_, ≥ 99.0%), l-ascorbic acid (≥99%), hydrochloric acid (37%), and 1-decanol (*n-*decanol, 98%) were purchased from Merck. Milli-Q grade water (resistivity 18.2 MΩ cm at 25 °C) was used in all experiments.

### Synthesis of Au NRs

The synthesis of the seeds was carried out according to a modified protocol described by González-Rubio et al.^47^

#### Gold Seeds

To prepare the growth solution, 9.111 g of CTAB (50 mM) and 870.5 mg (11 mM) of n-decanol were added to 500 mL of water and stirred at approximately 60 °C for 30–60 min.

The mixture was then cooled down to 30 °C, and 250 μL of a 0.05 M HAuCl_4_ solution was added to 25 mL of the *n*-decanol/CTAB solution in a 50 mL glass beaker.

The resulting mixture was stirred at 300 rpm for 5 min. Next, 125 μL of a 0.1 M ascorbic acid solution was added, causing the orange-yellow solution to slowly turn colorless. At this point, a freshly prepared 20 mM NaBH_4_ solution was injected (one shot) under stirring at 1000 rpm and 30 °C. The injection resulted in brownish-yellow solutions, and the seed solutions were aged for at least 60 min at 30 °C before use. It is important to note that the dimensions of the PTFE plain magnetic stirring bar (30 × 6 mm^2^) used in the stirring process can strongly affect the quality of the seeds.^47^

#### Synthesis of Anisotropic Seeds

In a typical synthesis, 1000 μL of 0.05 M HAuCl_4_, 800 μL of 0.01 M AgNO_3_, 7 mL of 1 M HCl, and 1300 μL of 0.1 M ascorbic acid were added under vigorous stirring to 100 mL of a 50 mM CTAB and 13.5 mM *n*-decanol solution at exactly 25 °C. Once the solution became colorless, 6 mL of the seed solution was added under stirring, and the mixture was left undisturbed for at least 4 h. The solution changed from colorless to dark brownish gray, and the recorded longitudinal LSPR was located at 725–730 nm. The small anisotropic seeds were centrifuged at 14,000–15,000 rpm for 60 min in 2 mL tubes. The precipitate was collected, redispersed with 10 mL of a 10 mM CTAB solution, and centrifuged twice under the same conditions. The final Au^0^ concentration was fixed to 4.65 mM (Abs400 nm: 10, optical path of 1 cm).

#### Growth of Au NRs

To synthesize nanorods, a 50 mL n-decanol/CTAB solution (11 and 50 mM, respectively) was prepared in a 50 mL glass vial. Then, 500 μL of 0.05 M HAuCl_4_ and 1250 μL of 0.01 M AgNO_3_ were added, followed by the addition of 400 μL of 0.1 M ascorbic acid and 3500 μL of 1 M HCl solutions under vigorous stirring. The growth was initiated by adding 250 μL of the anisotropic seed solutions (Abs at 400 nm = 1, optical path: 0.1 cm, [Au] = 4.75 mM, [CTAB] = 10 mM). After stirring the mixture for 30 s, it was left undisturbed for 3 h at 25 °C. The resulting nanorods were washed by centrifugation in 50 mL tubes at 6000 rpm for 30 min. The precipitate was redispersed in 2 mL of a 1 mM CTAB solution, and the concentrated Au NRs were centrifuged twice under the same conditions.

### Synthesis of Au@PdAg NRs

The seeded growth method with modifications,^4^ was used to prepare Au@PdAg NRs. To a freshly prepared 50 mM CTAB mixture solution in a 10 mL glass vial were added 0, 10, 80, 160, and 320 μL of 0.001 M AgNO_3_ and 1000 μL of 0.05 M Na_2_PdCl_4_ with stirring. 61 mL of the Au NR solution (Abs at 400 nm = 0.4, optical path: 0.1 cm, [Au] = 4.53 mM, [CTAB] = 50 mM) was then injected under magnetic stirring for homogenization. Next, 13 μL of a 0.1 M ascorbic acid solution was added with vigorous stirring (ca. 1000 rpm). The mixture was heated in a water bath at 65 °C, and stirring (300–500 rpm) was stopped after 10 min. The growth solution was left undisturbed in the water bath at 65 °C for at least 12 h. The resulting Au@PdAg NRs were then centrifuged in 2 mL tubes at 4000 rpm for 30–60 min, and the precipitate was redispersed with 2 mL of a 2 mM CTAB solution. The concentrated Au@PdAg NRs were centrifuged twice under the same conditions and stored in a 2 mM CTAB solution.

### Transmission Electron Microscopy and EDX Analysis

Low-magnification TEM images were obtained by using a JEOL JEM-1400 PLUS transmission electron microscope operating at an acceleration voltage of 200 kV. EDX elemental analyses were performed using a detector (Super-X) with 0.23 sr solid angle at the same microscope.

### Electron Tomography in HAADF-STEM and EDX Mode

A ThermoFisher Tecnai Osiris electron microscope operated at 200 kV in HAADF-STEM mode at typical beam currents of 50 pA and a Super-X detector at the same microscope at typical beam currents of 150 pA (acquisition times of 10 min) were used to acquire the STEM images and EDX-based elemental maps. A previous approach was followed, as detailed elsewhere, for EDX tomography:^72,73^ EDX maps were acquired every 10° and quantified according to the ζ-factor method once HAADF-STEM tomography series in a tilt range of ±75° with an increment of 3° were obtained. EDX voxel line scan intensity values extracted from the 3D reconstruction showed a small Pd signal originating from the core of the nanoparticle for nonirradiated samples.^74^ This outcome was unlikely given the multistep synthetic method used. To confirm that this signal was a consequence of background noise and was within an acceptable error range, a specialized quantitative reconstruction technique was applied. A 3D mask was generated from the HAADF-STEM tomography reconstruction and subsequently applied to the corresponding EDX data sets. A more detailed description of this advanced quantitative reconstruction technique can be found in the following publication.^2^ Upon 3D mask application, the remaining Pd signal was isolated to the shell, with no Pd signal found in the core. In contrast to nonirradiated nanoparticles, equivalent EDX voxel line scans of Au@Pd nanoparticles after fs-irradiation showed a higher Pd signal at the core, most notably at the Au–Pd interface. The higher Pd signal and overlapping signals of Au and Pd at the interface suggest elemental intermixing of the core–shell structure upon irradiation.

### UV–Vis-NIR Spectra

All experiments were carried out using a Varian Cary 5G at 298 K and quartz cuvettes with optical paths of 1 cm.

### Irradiation Experiments

Au@PdAg NRs with a broad longitudinal LSPR centered at ca. 800 nm were irradiated with 50 fs 804 nm laser pulses generated with an amplified Ti:sapphire laser system (Spectra-Physics). Samples were irradiated in quartz cuvettes (4 mL volume, 200–2500 nm spectral range) with an optical path of 1 cm and a fixed volume of 2.5 mL (under constant stirring at 300 rpm using a magnetic bar at room temperature). Two irradiation regimes were applied: focused and nonfocused beam irradiation. For the latter, fluence control was performed with a continuously variable neutral density filter wheel. In this case, the selected irradiation fluence was 10 J/m^2^ with a beam diameter of about 1 cm and irradiation times of 25 min. In the focused beam regime, the laser beam was focused with a 25 cm focal length silica lens and the sample was placed 10 cm from the lens to avoid damage to the cuvette. In this case, the laser fluence was 70 J/m^2^ with a beam diameter of about 6 mm and an irradiation time of 25 min.

### X-ray Absorption Spectroscopy (XAS)

XAS measurements were conducted in the X-ray absorption near-edge structure (XANES) regime on samples at the BL22 CLÆSS beamline of the ALBA synchrotron facility in Cerdanyola del Vallès, Spain.^75^ The measurements were taken at room temperature in fluorescence mode at the Au *L*_3_-edge (11919 eV). An Au metal foil was used for calibration. The XAS analysis was carried out using the Athena.^76^

### Optical Simulations

Optical response was calculated using the method of finite differences in the time domain (FDTD), as implemented in the free software package MEEP.^77^ In this method, Maxwell equations are solved by a second-order approximation. Space is divided into a discrete grid, the Yee grid,^78^ and the fields are evolved in time using discrete time steps. A schematic representation of the geometry used for the calculation is shown in Figure S8. Simulations were performed for nanostructures (Au NR with lengths and diameters of 66 and 18 nm, respectively, surrounded by a cage of Pd with a width of 31 nm and a height of 80 nm) oriented along the three Cartesian axes. In all calculations, we employed a spatial resolution of 0.5 nm. For the refractive index, we applied the bulk values of Au and Pd fixed by Johnson and Christy,^79^ using a Drude term and five Lorentzians.^80^ The refractive index of the surrounding medium was fixed at 1.33 (water). In Figure S8, *d*_1_ and *d*_2_ are, respectively, the rod diameter and the box side. Likewise, *L*_1_ and *L*_2_ represent the lengths of the rod and the box, respectively. The edges of the box were rounded with a radius of 2 nm to make it more like the experimental structures.

## Supporting Information Available

Synthetic details and characterization techniques; fs laser irradiation experiments; HAADF-STEM tomography experiments and corresponding representative movies (PDF)
