# Supplementary material for: From Multi- to Single-Hollow Trimetallic Nanocrystals by Ultrafast Heating
Source: Chem Mater. 2023 Nov 6;35(22):9603–12. doi: 10.1021/acs.chemmater.3c01698 (PMC10687867; doi:10.1021/acs.chemmater.3c01698)
Supplement: Supplementary file 1 — cm3c01698_si_001.pdf [file cm3c01698_si_001.pdf]

SUPPLEMENTARY INFORMATION

From Multi- to Single-Hollow Trimetallic Nanocrystals by Ultrafast Heating

Vanesa Manzaneda-González,[†,‡] *Kellie Jenkinson,*[†,§] *Ovidio Peña-Rodríguez,*[⊥,ǂ] *Olivia Borrell-Grueiro,*[‡] *Sergio Triviño-Sánchez,*[‡] *Luis Bañares,*[‡,#] *Elena Junquera,*[‡] *Ana Espinosa,*[ø] *Guillermo González-Rubio,*[‡,*] *Sara Bals,*[§,*] *Andrés Guerrero-Martínez*[‡,*]

[‡]Departamento de Química Física, Universidad Complutense de Madrid, Avenida Complutense s/n, 28040 Madrid, Spain.

[§]EMAT, University of Antwerp, Groenenborgerlaan 171, B-2020 Antwerp, Belgium.

[⊥]Instituto de Fusión Nuclear "Guillermo Velarde", Universidad Politécnica de Madrid, José Gutiérrez Abascal 2, E-28006 Madrid, Spain.

[ǂ]Departamento de Ingeniería Energética, ETSII Industriales, Universidad Politécnica de Madrid, José Gutiérrez Abascal 2, E-28006 Madrid, Spain.

[#] Instituto Madrileño de Estudios Avanzados en Nanociencia (IMDEA-Nanoscience), Cantoblanco, 28049 Madrid, Spain.

[ø] Instituto de Ciencia de Materiales de Madrid, Consejo Superior de Investigaciones Científicas, calle Sor Juana Inés de la Cruz 3, Madrid 28049, Spain.

[†] V.M.-G. and K.J. contributed equally to this work.

*Corresponding authors. Email: ggrubio@ucm.es (G.G.-R.), sara.bals@uantwerpen.be (S.B.), aguerrero@quim.ucm.es (A.G.-M.)

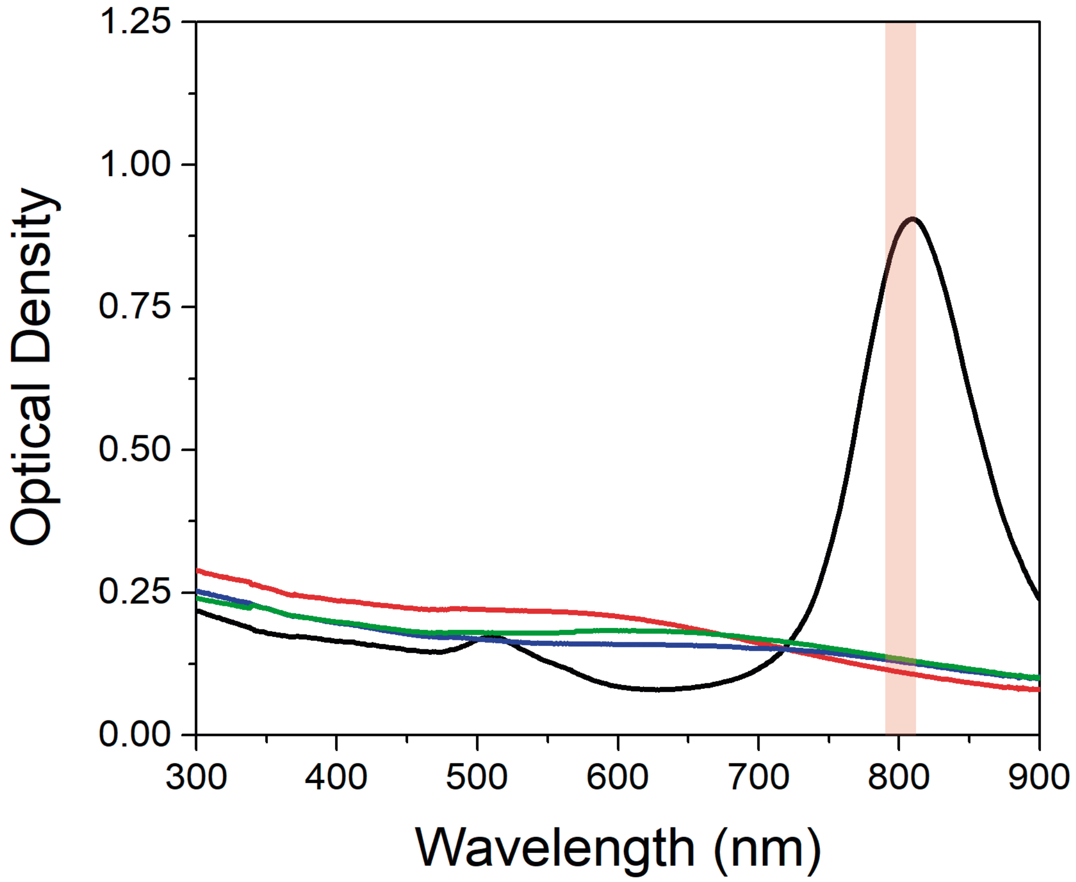

Figure S1. UV-Vis-NIR spectra of Au NR (black), Au@Pd NR (red), $Au_{13}@Pd_{76}Ag_{11}$ NR (blue) and $Au_{12}@Pd_{69}Ag_{19}$ NR (green). The red vertical band represents the 800 nm 50-fs pulsed laser used for the irradiation experiments.

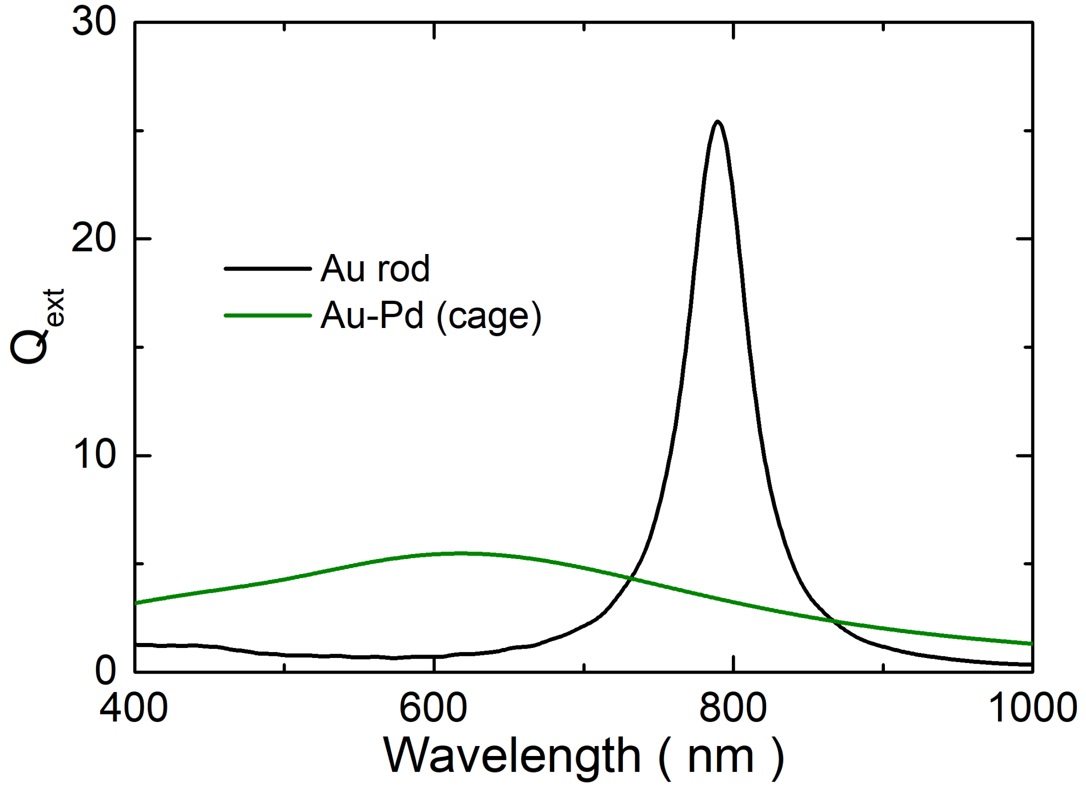

Figure S2. Extinction efficiency (Q_{ext}) for a gold nanorod (black line) and a Au@Pd NR (green line), calculated with the longest axis of the structure parallel to an *x*-polarized plane wave.

.

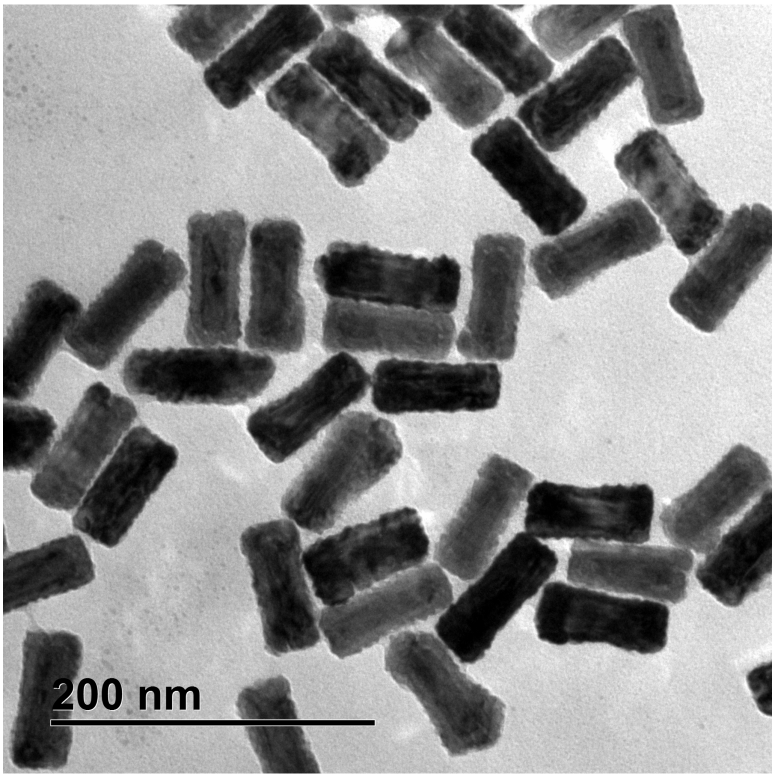

Figure S3. Low-magnification TEM image of $Au_{15}@Pd_{75}Ag_{10}$ NRs synthesized in the presence of CTAC.

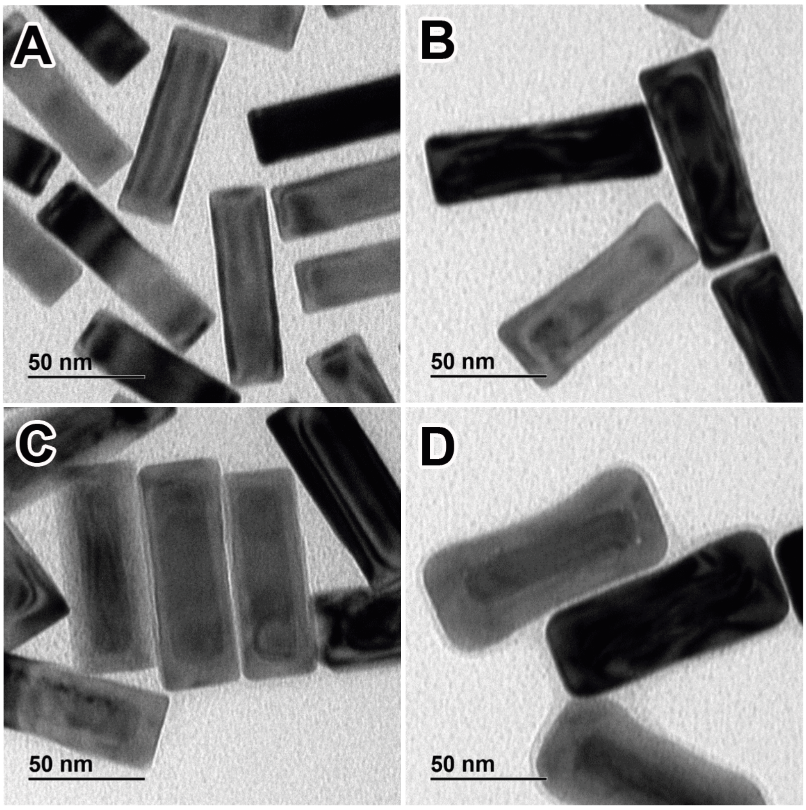

Figure S4. Au NR@Pd structures obtained in the absence of Ag**.** TEM images at different times during the growth process of Au@Pd NRs: 10 min (A), 20 min (B), 45 min (C), and 12h (D).

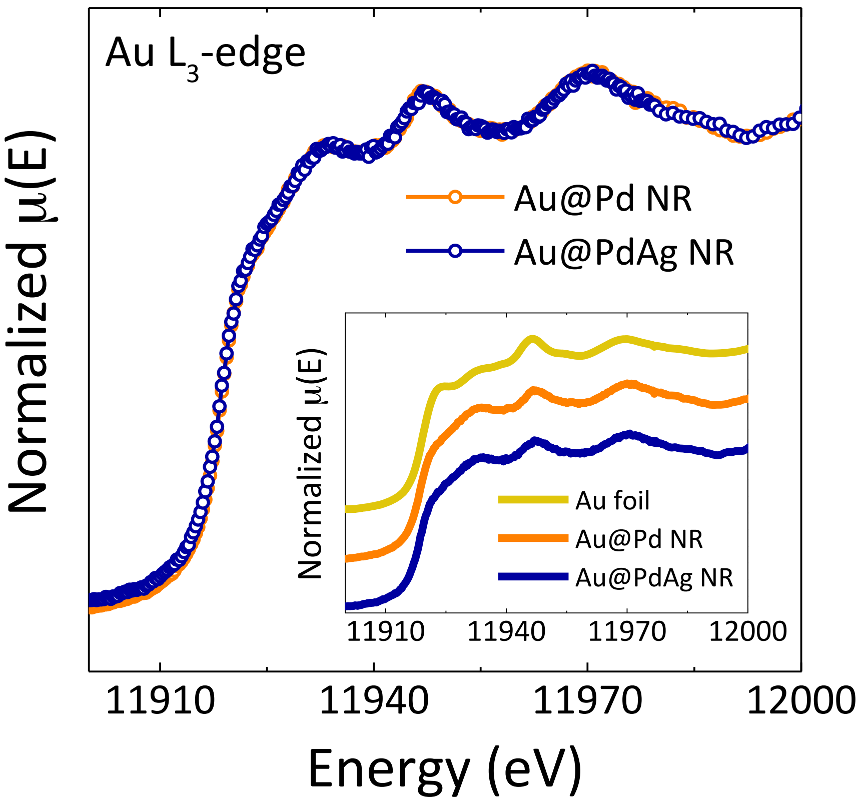

Figure S5. XANES spectra at the Au L_3-edge of Au@Pd NRs and Au@PdAg NRs samples. Inset: XANES spectra of samples compared with Au metallic foil.

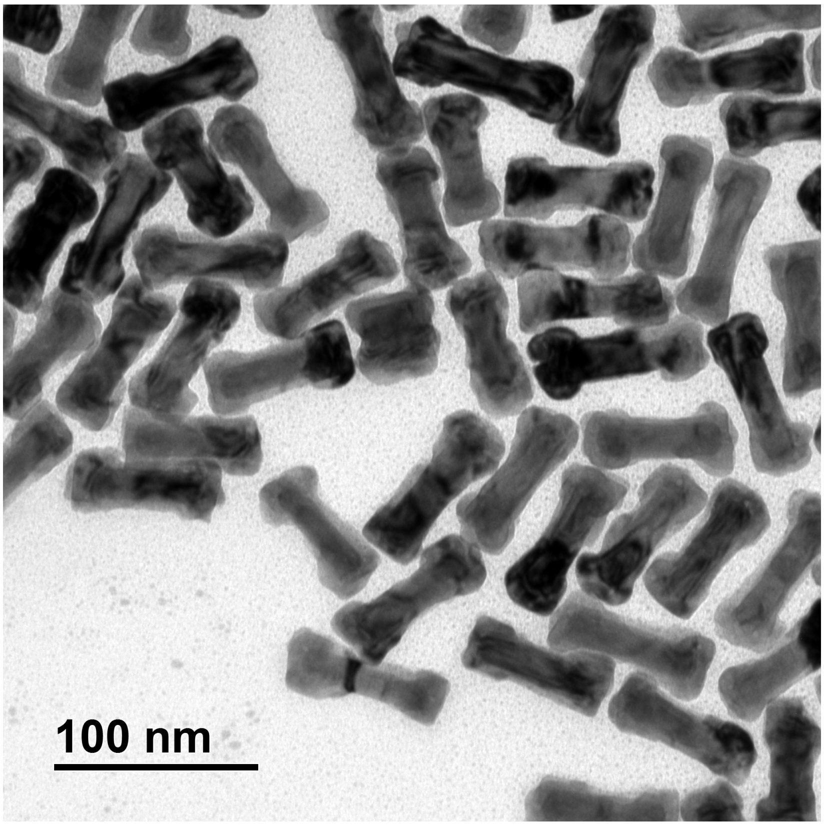

Figure S6. Low-magnification TEM image of Au_{15}@$Pd_{80}Ag_5$ NRs.

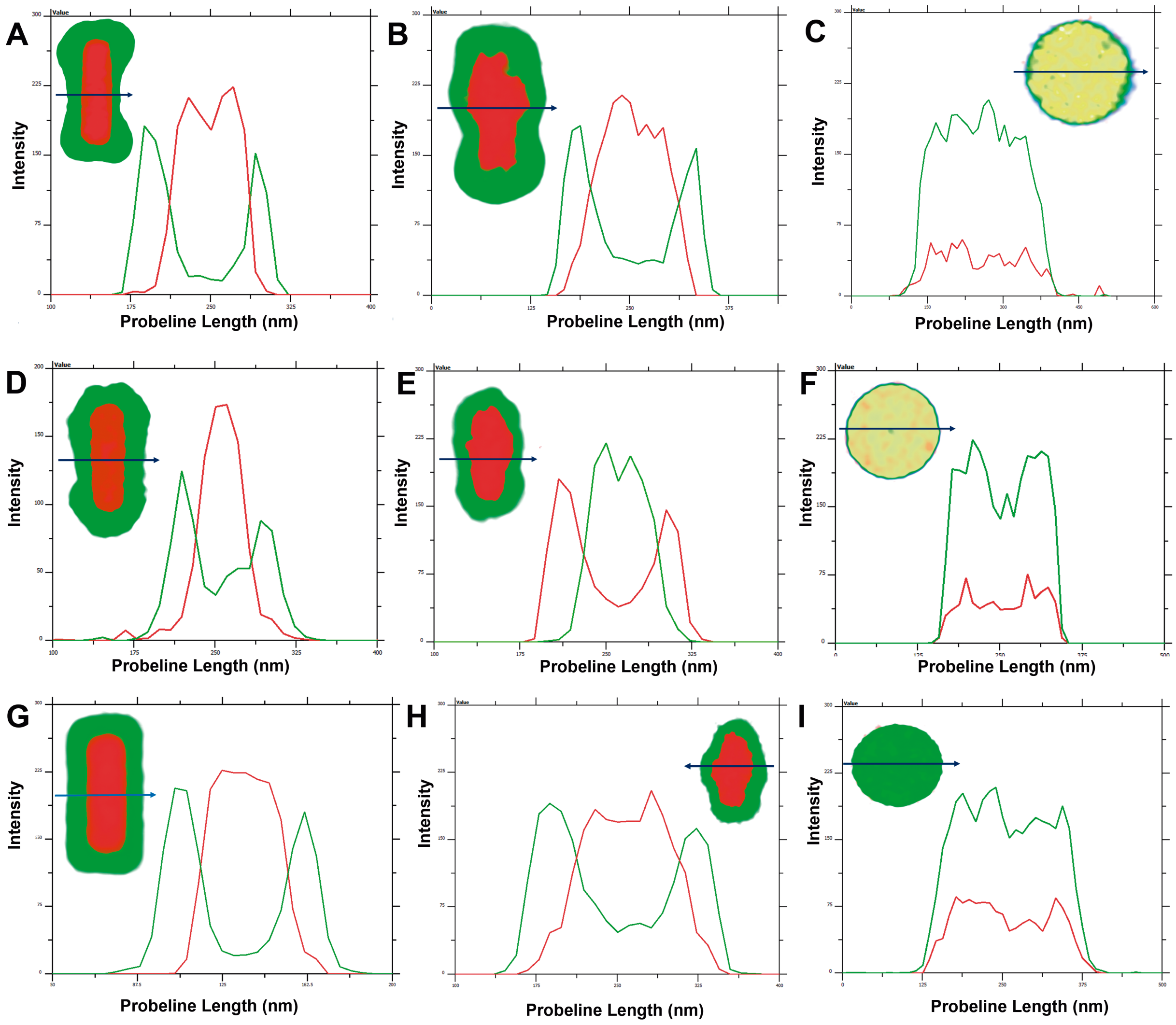

Figure S7. Elemental distribution by voxel line scan through the centre of the reconstructed $Au_{13}@Pd_{76}Ag_{11}$ NR (A-C), $Au_{12}@Pd_{69}Ag_{19}$ NR (D-F), and Au@Pd NR (G-I) before (A,D,G) and after irradiation with fs-laser pulses at a fluence of 10 J/m^2 (B,E,H) and 70 J/m^2 (C,F,I).

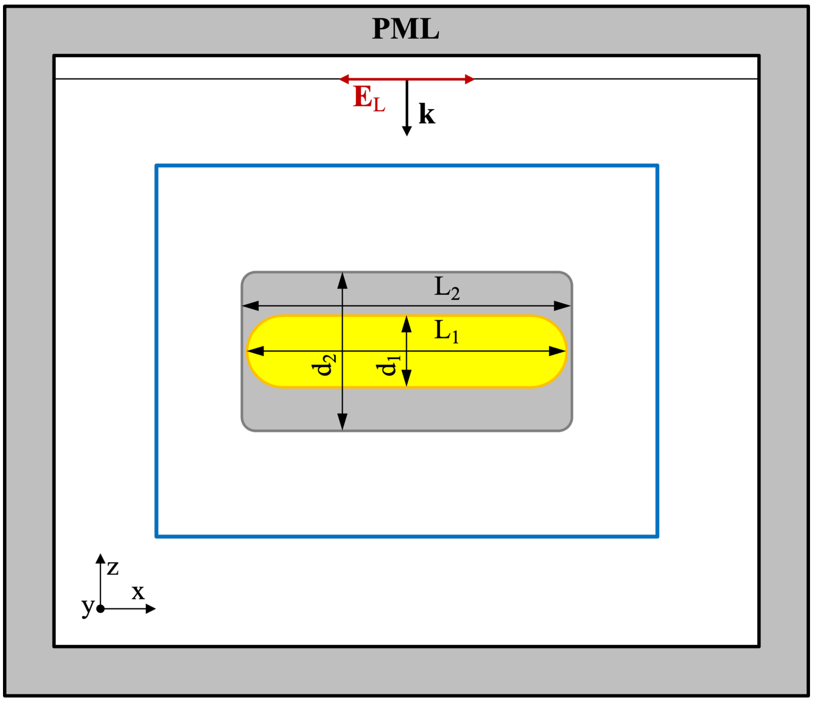

Figure S8. 2D schematic representation of the 3D model used to simulate the optical response of the core–shell nanoparticles. An x-polarized plane wave impinges over the nanostructure and the flux is measured on all the faces of a parallelepiped containing the whole nanostructure (the blue rectangle) to determine the extinction efficiency (Q_{ext}). The simulation is performed with the plasmonic nanoparticle aligned along the x Cartesian axis, to determine the longitudinal plasmon mode. The simulation area is surrounded by a perfectly matched layer (PML) to mimic an infinite space.

SUPPLEMENTARY MOVIES

Movie_S1. Electron tomography reconstruction of a $Au_{13}@Pd_{76}Ag_{11}$ NR.

Movie_S2. Electron tomography reconstruction of a $Au_{13}@Pd_{76}Ag_{11}$ NR where it is possible to observe the internal voids

Movie_S3. Animated orthoslices of a $Au_{13}@Pd_{76}Ag_{11}$ NR where it is possible to locate the internal voids.

Movie_S4. Electron tomography reconstruction of a $Au_{12}@Pd_{69}Ag_{19}$ NR.

Movie_S5. Electron tomography reconstruction of a $Au_{12}@Pd_{69}Ag_{19}$ NR where it is possible to observe the internal voids

Movie_S6. Animated orthoslices of a $Au_{12}@Pd_{69}Ag_{19}$ NR where it is possible to locate the internal voids.

Movie_S7. Electron tomography reconstruction of a $Au_{13}@Pd_{76}Ag_{11}$ NR irradiated for 25 min with fs-laser pulses at a fluence of 10 J/m^2.

Movie_S8. Electron tomography reconstruction of a $Au_{13}@Pd_{76}Ag_{11}$ NR irradiated for 25 min with fs-laser pulses at a fluence of 10 J/m^2 where it is possible to observe the internal voids

Movie_S9. Animated orthoslices of a $Au_{13}@Pd_{76}Ag_{11}$ NR irradiated for 25 min with fs-laser pulses at a fluence of 10 J/m^2 where it is possible to locate the internal voids.

Movie_S10. Electron tomography reconstruction of a $Au_{12}@Pd_{69}Ag_{19}$ NR irradiated for 25 min with fs-laser pulses at a fluence of 10 J/m^2.

Movie_S11. Electron tomography reconstruction of a $Au_{12}@Pd_{69}Ag_{19}$ NR irradiated for 25 min with fs-laser pulses at a fluence of 10 J/m^2 where it is possible to observe the internal voids

Movie_S12. Animated orthoslices of a $Au_{12}@Pd_{69}Ag_{19}$ NR irradiated for 25 min with fs-laser pulses at a fluence of 10 J/m^2 where it is possible to locate the internal voids.

Movie_S13. Visualization of the 3D elemental mapping of a $Au_{13}@Pd_{76}Ag_{11}$ NR irradiated for 25 min with fs-laser pulses at a fluence of 10 J/m^2 where the Au NR (red) and PdAg shell (green) can be readily distinguished.

Movie_S14. Animated orthoslices the 3D elemental mapping of a $Au_{13}@Pd_{76}Ag_{11}$ NR irradiated for 25 min with fs-laser pulses at a fluence of 10 J/m^2 where Au (red) and PdAg (green) can be readily distinguished.

Movie_S15. Visualization of the 3D elemental mapping of a $Au_{12}@Pd_{69}Ag_{19}$ NR irradiated for 25 min with fs-laser pulses at a fluence of 10 J/m^2 where the Au NR (red) and PdAg shell (green) can be readily distinguished.

Movie_S16. Animated orthoslices of the 3D elemental mapping of a $Au_{12}@Pd_{69}Ag_{19}$ NR irradiated for 25 min with fs-laser pulses at a fluence of 10 J/m^2 where Au (red) and PdAg (green) can be readily distinguished.

Movie_S17. Electron tomography reconstruction of a $Au_{13}@Pd_{76}Ag_{11}$ NR irradiated for 25 min with fs-laser pulses at a fluence of 70 $J/m^{2.}$

Movie_S18. Animated orthoslices of a $Au_{13}@Pd_{76}Ag_{11}$ NR irradiated for 25 min with fs-laser pulses at a fluence of 70 J/m^2 where it is possible to locate the internal void.

Movie_S19. Animated orthoslices of a $Au_{13}@Pd_{69}Ag_{19}$ NR irradiated for 25 min with fs-laser pulses at a fluence of 70 J/m^2 where it is possible to observe the internal void.

Movie_S20. Visualization of the 3D elemental mapping of a $Au_{13}@Pd_{76}Ag_{11}$ NR irradiated for 25 min with fs-laser pulses at a fluence of 70 J/m^2 where the AuPdAg alloy formation is revealed.

Movie_S21. Electron tomography reconstruction of a $Au_{12}@Pd_{69}Ag_{19}$ NR irradiated for 25 min with fs-laser pulses at a fluence of 70 J/m^2.

Movie_S22. Electron tomography reconstruction of a $Au_{12}@Pd_{69}Ag_{19}$ NR irradiated for 25 min with fs-laser pulses at a fluence of 70 J/m^2 where it is possible to observe the internal void.

Movie_S23. Animated orthoslices of a $Au_{12}@Pd_{69}Ag_{19}$ NR irradiated for 25 min with fs-laser pulses at a fluence of 70 J/m^2 where it is possible to observe the internal void.

Movie_S24. Visualization of the 3D elemental mapping of a $Au_{12}@Pd_{69}Ag_{19}$ NR irradiated for 25 min with fs-laser pulses at a fluence of 70 J/m^2 where the AuPdAg alloy formation is revealed.

Movie_S25. Electron tomography reconstruction of a Au@Pd NR

Movie_S26. Electron tomography reconstruction of a Au@Pd NR where it is possible to observe the Au NR core and the absence of internal voids

Movie_S27. Visualization of the 3D elemental mapping of a Au@Pd NR (Au: red, Pd:green).

Movie_S28. Animated orthoslices of the 3D elemental mapping of a Au@Pd NR (Au: red, Pd:green).

Movie_S29. Electron tomography reconstruction of a Au@Pd NR irradiated for 25 min with fs-laser pulses at a fluence of 10 J/m^2

Movie_S30. Electron tomography reconstruction of a Au@Pd NR irradiated for 25 min with fs-laser pulses at a fluence of 10 J/m^2 where it is possible to observe the absence of internal voids

Movie_S31. Visualization of the 3D elemental mapping of a Au@Pd NR irradiated for 25 min with fs-laser pulses at a fluence of 10 J/m^2 (Au: red, Pd:green).

Movie_S32. Animated orthoslices of the 3D elemental mapping of a Au@Pd NR irradiated for 25 min with fs-laser pulses at a fluence of 10 J/m^2 (Au: red, Pd:green).

Movie_S33. Electron tomography reconstruction of a Au@Pd NR irradiated for 25 min with fs-laser pulses at a fluence of 70 J/m^2

Movie_S34. Electron tomography reconstruction of a Au@Pd NR irradiated for 25 min with fs-laser pulses at a fluence of 70 J/m^2 where it is possible to observe the absence of internal voids

Movie_S35. Visualization of the 3D elemental mapping of a Au@Pd NR irradiated for 25 min with fs-laser pulses at a fluence of 70 J/m^2 (Au: red, Pd:green).
